# Impact of a nurse led telephone intervention on satisfaction and health outcomes of children with inflammatory rheumatic diseases and their families: a crossover randomized clinical trial

**DOI:** 10.1186/s12887-017-0926-5

**Published:** 2017-07-17

**Authors:** Anne-Sylvie Ramelet, Béatrice Fonjallaz, Laura Rio, Sandra Zoni, Pierluigi Ballabeni, Joachim Rapin, Christophe Gueniat, Michaël Hofer

**Affiliations:** 1 Institute of Higher Education and Research in Healthcare-IUFRS, University of Lausanne, University Hospital of Lausanne, Rte de la Corniche 10, 1011 Lausanne, Switzerland; 2 Geneva League for Rheumatology, La ligue Genevoise contre le Rhumatisme, Rue Merle d’Aubigné 22, 1207 Geneva, Switzerland; 3 Pediatric Medico-chirurgical Department of University Hospital of Lausanne, CHUV, Rue du Bugnon 21, 1011 Lausanne, Switzerland; 4 Haute Ecole de Santé Vaud (HESAV), University of Applied Sciences and Arts Western Switzerland, Rte de la Corniche 10, 1011 Lausanne, Switzerland

**Keywords:** Telehealth, Telenursing, Patient satisfaction, Pediatrics, Rheumatology, Symptom management

## Abstract

**Background:**

Children suffering from rheumatic disease are faced with multidimensional challenges that affect their quality of life and family dynamics. Symptom management and monitoring of the course of the disease over time are important to minimize disability and pain. Poor disease control and anticipation of the need for treatment changes may be prompted by specialist medical follow-up and regular nurse-led consultations with the patient and families, in which information and support is provided. The purpose of this study was to evaluate the impact of a nurse-led telephone intervention or Telenursing (TN) compared to standard care (SC) on satisfaction and health outcomes of children with inflammatory rheumatic diseases and their parents.

**Methods:**

A multicentered, randomized, longitudinal, crossover trial was conducted with pediatrics outpatients newly diagnosed with inflammatory rheumatic diseases. Participants were randomly assigned to two groups TN and SC for 12 months and crossed-over for the following 12 months. TN consisted of providing individualized affective support, health information and aid to decision making. Satisfaction (primary outcome) and health outcomes were assessed with the Client Satisfaction Questionnaire-8 and the Juvenile Arthritis Multidimensional Assessment Report, respectively. A mixed effect model, including a group x time interaction, was performed for each outcome.

**Results:**

Satisfaction was significantly higher when receiving TN (OR = 7.7, 95% CI: 1.8–33.6). Morning stiffness (OR = 3.2, 95% CI: 0.97–7.15) and pain (OR = 2.64, 95% CI: 0.97–7.15) were lower in the TN group. For both outcomes a carry-over effect was observed with a higher impact of TN during the 12 first months of the study. The other outcomes did not show any significant improvements between groups.

**Conclusion:**

TN had a positive impact on satisfaction and on morning stiffness and pain of children with inflammatory rheumatic diseases and their families. This highlights the importance of support by specialist nurses in improving satisfaction and symptom management for children with inflammatory rheumatisms and their families.

**Trial registration:**

ClinicalTrial.gov identifier: NCT01511341 (December 1st, 2012).

## Background

Pediatric rheumatic diseases comprise a large group of inflammatory and non-inflammatory diseases of the locomotion system and are considered as an important pediatric chronic illness worldwide. In the US, 300′000 children are affected by rheumatic diseases; it is 100′00 more than those with juvenile diabetes [1]. In Switzerland, the annual incidence rate was 40.6 new patients per 100,000 children, with 56.8/100,000 in the Canton of Vaud (Western Switzerland); about two thirds were diagnosed with an inflammatory disease [2].

Juvenile idiopathic arthritis (JIA) is the most common form of rheumatic diseases [3]. Children and adolescents with JIA commonly experience chronic pain, decreased functional ability, impaired physical development, decreased overall well-being and quality of life, and emotional, social, and school functioning when compared to healthy individuals [4, 5]. Currently, there is no cure and heavy treatments involving medication such as anti-inflammatory drugs, corticosteroid injections, and TNF alpha blockers; surgery and occupational therapy. Those treatments are challenging for children and their families. Families have to learn how to adjust to their child’s needs, and also how to mobilize their resources to maintain their own health and positive mental images, and manage their uncertainty, anxiety, and distress [6, 7]. In our centre, unpublished pilot data showed that families of children with rheumatic disease were not entirely satisfied (median score of 26.8 ± 3.4/32), especially due to the lack of contacts with health professionals between follow-up medical visits.

Caring for children with rheumatic chronic disease involves a multidisciplinary approach. In addition to medical care, nurses play a key role in supporting the specialist team caring for these patients, recognizing poor disease control and the need for changes in treatment, providing information on treatment options and how to access additional support. Nurses also ensure the link between medical practitioner, other health providers, and family. These types of nursing care can be provided via telephone, so called Telenursing (TN) [8]. Impact of TN has mostly been studied in adult patients with chronic disease and showed decreased hospitalization rates, emergency department visits, exacerbations, hospitalizations number, and mean duration of bed days [9–13]. In the pediatric setting, the literature review highlighted the paucity of studies demonstrating strong evidence of the benefits of TN. In some studies involving children with complex special healthcare needs, TN interventions were more geared towards alleviating physicians’ workload and compensating for subspecialist shortage [14–16]. In studies targeting parents and children directly, TN was done via a Helpline for parents of children with congenital anomalies [17], or suffering from gastroenteritis [18] or via Smartphone text for mothers and children undergoing tonsillectomy [17–19]. To the best of our knowledge, no studies testing the effect of TN in pediatric ambulatory care for children with chronic disease have been published so far. This study aims to test the impact of a nursing consultation via telephone on health-related outcomes of patients and satisfaction of participants.

## Methods

### Study design

A randomized crossover, experimental longitudinal design was used in this study (see protocol published elsewhere) [20]. This article presents the quantitative component of this study.

### Setting and participants

The setting was a tertiary referral pediatric rheumatology outpatient clinic, serving all French-speaking cantons of Switzerland. Every year, about 110 new patients are admitted to the clinic; about 50 of them have chronic inflammatory rheumatic diseases.

The study participants were the designated users of the nursing telephone consultation, therefore included parents of children ≤11 years or children from 12 years of age. Children newly diagnosed (within 18 months prior to the enrolment date) with an inflammatory rheumatic disease, including JIA, connective tissue disease, and vasculitis and under the age of 16 at enrolment or their parents were eligible. Upon agreement to study participation, informed consent and witten assent were provided by parents and children (aged between 11 and 16), respectively. Potential participants that did not understand and speak French and/or had no access to a telephone were excluded.

### Recruitment and randomization procedures

The study and its amendment were approved by the Human Research Ethics Committee of the canton of Vaud, Switzerland on January 17, 2011 and March 28, 2011, respectively. Parents and patients who attended the pediatric rheumatology outpatient clinic between January 2010 and August 2012 and consented were enrolled in the study for a total of 24 months. Briefly, this study was a randomized, crossover trial, in which patients were their own control [21]. The intervention (TN) was evaluated against standard care (SC) with the same subjects. It is worth noting that TN was provided in addition to SC, thus all participants received SC for the whole duration of this study. Participants were randomized and allocated to group 1 or group 2 using a computer-generated simple block randomization to account for different level of severity of illness. Treatment allocation was in sealed numbered envelopes. Both groups received 12 months of TN and 12 months of SC, only the attribution order varied; (group 1 received TN first and then SC and group 2 received SC first and then TN).

### Theoretical framework and delivery of the intervention

The Cox’s Interaction Model of Client Health Behavior (IMCHB), which was developed to direct and document nursing evaluation and care and reach positive nursing intervention effects on health outcome, was used to guide this study [22]. The main objective of this nurse-led intervention was to ensure continuity of care for children and their families. TN provided by specialized nurses via telephone included provision of individualized health information, affective support and help in decision making. Two qualified specialist nurses with over five years of experience in adult and pediatric rheumatology were specifically trained (3 day course) in TN oral communication, strategies for questioning parents and adolescents, assessing the quality of interactions and aiding decision making for the TN. A two-part standardized form of telephone interviewing was developed for each TN consultation. The first part included description of the call, such as time, initiator and nature of the call, action/decision taken, and a brief summary of the conversation and planned action. The second part related to the intervention itself and included eight questions on: 1) everyday life, school and social, 2) treatment, 3) physiotherapy, 4) occupational therapy, 5) pain, 6) schedule, 7) administrative issues, 8) any additional topic that the respondent would like to discuss.

### Experimental group

As per cross-over design, all participants received the intervention (TN), either during the first 12 months or the last 12 months of the study. When in the TN group, participants attended a first face-to-face medical and nursing consultation at the start of TN (T0 for group 1; T12 for group 2). This visit allowed the TN nurse to introduce herself, explain how the telephone consultation would be carried out and get to know the child’s clinical, social and family situation. For the following 12 months, the participants received a monthly telephone call. In addition, the participating parent or child was given a telephone number to contact, when needed, the TN nurse on duty during normal office hours on week days.

### Control group

As per cross-over design, all participants included were part of the control group, either during the first 12 months or the last 12 months of the study. Participants in the control group received SC, in which medical management was provided by a pediatric rheumatologist mainly, but also by other specialists (occupational therapists) as determined by patients’ needs. When in the SC group, participants attended a face-to-face medical consultation only, at the start of SC (T12 for group 1, T0 for group 2). The medical consultation was repeated every three months and participants were followed and treated as per standard practice. Parents were also informed that they could call the outpatient clinic.

### Measures

The choice of data collection points and the study time span was based on theoretical and practical considerations [23]. Face-to-face consultation baseline data (demographics, health status, and satisfaction) were collected at T0. Demographic data about participants included age, gender, cultural background, marital status, occupation, education, language spoken at home, and types of treatment. Collection points occurred every three months for disease activity and health status assessment (T3, T6, T9, T12, T15, T18, T21 and T24) and every six months for satisfaction (T6, T12, T18 and T24).

### Outcomes

The study’s primary outcome was participants’ satisfaction (child/parent). Satisfaction was assessed using the Client Satisfaction Questionnaire-8 (CSQ-8), an 8-item version of the 18-item CSQ [24]. It is brief to administer, has good psychometric properties, and has been translated and validated in French. Each item of the CSQ-8 items is rated on a 4-point Likert-type scale giving a total score ranging between 8 (no satisfaction) and 32 (total satisfaction), a score ≥ 30 indicates satisfactory rating of satisfaction and a score < 30 a lack of satisfaction. Test comparison was the proportion of subjects who were satisfied in each group as well as changes in satisfaction scores within groups (between T0 and T12).

Secondary outcomes were clinical health status measurements performed every three months as per standard practice, using the Juvenile Arthritis Multidimensional Assessment Report (JAMAR) French version, of which original version was translated and validated [25–27]. The JAMAR includes 15 self-reported measures that assess well-being, pain, functional status, health-related quality of life, morning stiffness, disease activity, disease status and course, joint disease, extraarticular symptoms, side effects of medications, therapeutic compliance, and satisfaction with illness outcome. For children too young to self-report, the parent version of the JAMAR was used. In this study, the items of interest were: (1) Assessment of functional ability through a 15-item questionnaire, in which the ability of the child to carry out daily living activities is scored: 0 = without difficulty, 1 = with difficulty, 2 = unable to do. A total score of 0 was considered as no difficulty in functional ability and a total score of ≥1 was considered as having some difficulty; (2) Rating of the intensity of child’s pain on a 21-point visual analogue scale (VAS) (0 = no pain; 10 = extreme pain). A total score of ≤3 was considered as no pain and a total of ˃3 was considered as having pain; (4) Assessment of morning stiffness was a “yes-no” item; (5) Assessment of extraarticular symptoms was two “yes- no” questions assessing fever and rash; (7) Rating of disease status at the time of the visit as remission, continued activity, or relapse; (8) Rating of disease course from previous visit as much improved, slightly improved, stable/unchanged, slightly worsened or much worsened (improvement of disease status was assessed when participants answered “remission” at item 7 and “much improved” or “slightly improved” at item 8); (14) Assessment of health-related quality of life was performed through a 10-item questionnaire having two dimensions, physical health and psychosocial health, composed of 5 items each. The responses were “never” (score = 0), “sometimes” (score = 1), “most of the time” (score = 2), and “all the time” (score = 3). Separate scores for the physical and psychosocial subscales can also be calculated. A total score of 0 was considered as no difficulty in quality of life and a total of ≥1 was considered as having difficulty in quality of life. Same quotation was applied to the subscales.

The study’s secondary comparisons were the proportion of subjects that had: i) no morning stiffness; ii) no pain; iii) no difficulty in functional capacity; iv) in remission; v) no difficulty in physical quality of life and vi) no difficulty in psychosocial quality of life in both groups.

### Statistical analysis

Sample size and power were predicted based on the number of newly diagnosed children with inflammatory rheumatic diseases admitted to the study hospital’s paediatric rheumatology outpatients 2008 (*N* = 48). We anticipated that around 70 children would be admitted to the clinic in the 18-month screening period prior to enrolment into the study and considered that 80% of patients/parents would give consent to their participation (*N* = 56).

A power analysis was calculated based on the number of participants expected to complete the study, not the number recruited initially. For 50% difference in the proportion of subjects with a satisfaction score ≥ 30 (cut-off score) between the two groups, 23 subjects per group was required to reach a power level of .90 for an alpha level of .05 (two-sided test). To compensate for an expected attrition rate of 20%, we aimed to recruit 28 subjects in each group (total of 56 subjects).

An intention to treat analysis was performed. Random intercept mixed effect linear models were used for continuous outcomes and random intercept logistic mixed models for binary outcomes. The models tested the effect of treatment (TN or SC), period (year) and the interaction between treatment and period. Prior to data analyses, data were screened for data file’s accuracy, missing data, outliers, and distribution [28]. Data analyses were performed using Stata version 13 software (StataCorp LP, College Station, TX, USA).

## Results

### Participant flow

**Fig. 1:**
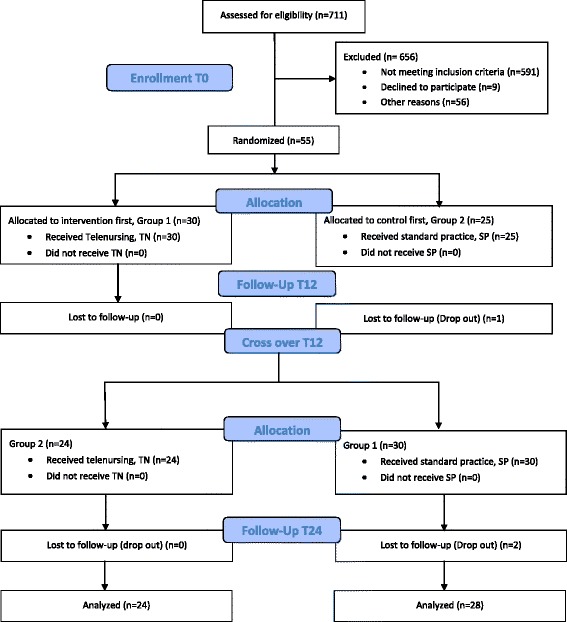
Study enrollment and flow

Figure 1 summarizes the recruitment and group’s allocation. Of 711 children initially screened, 120 were eligible and 55 (46%) consented to participate and were included. Participants were randomized and allocated to group 1 that received TN the first 12 months and then SC (*n* = 30) or group 2 that received SC first and then TN (*n* = 25). After 12 months, one participant of group 2 withdrew from the study. After 24 months two additional participants withdrew from group 1.

### Baseline data

Table 1 shows demographics of respondents at baseline. Responding children (*n* = 24, females 58.3%) had a mean age of 13.1 years, most of them were born in Switzerland and were still in school (87.5%). Responding parents (*n* = 31, 96.8% females) were mostly Swiss (71%), working (90.4%) and married (74.2%). Table 2 shows children clinical characteristics with the majority of them diagnosed with some form of JIA (70.5%), predominantly with juvenile enthesitis-related arthritis (ERA) (29%) or with oligoarticular JIA (27%). Other diagnosis included; uveitis (5.4%), chronic osteomyelitis (3.6%), chronic infantile neurological cutaneous articular (CINCA) syndrome (1.8%), lupus (1.8%), Crohn’s disease (1.8%), Behçet syndrome (1.8%), auto-inflammatory disease (1.8%) and juvenile dermatomyositis (JDM) (1.8%), with a few undetermined conditions (7.3%). Disease severity was assessed, by the treating physician using the Juvenile Arthritis Disease Activity Score (JADAS) as per standard practice and showed that most children had mild to moderate condition (90%) versus severe (10%).

**Table 1:** Baseline demographics of the study sample

	Children			Parents	
	Group 1	Group 2		Group 1	Group 2
	TN → SC ^a^	SC → TN ^a^		TN → SC ^a^	SC → TN ^a^
	(*n* = 14)	(*n* = 10)		(*n* = 16)	(*n* = 15)
Sex, female	8 (57%)	6 (60%)	Sex, female	15 (94%)	15 (100%)
Country of birth			Nationality		
Switzerland	12 (86%)	8 (80%)	Swiss	10 (63%)	12 (80%)
Europe	1 (7%)	2 (20%)	European	6 (37%)	2 (13%)
United States	1 (7%)	0	African	0	1 (7%)
Education			Professional activity		
Primary School ^b^	4 (29%)	6 (60%)	Commercial	4 (25%)	5 (33%)
Secondary School ^b^	8 (57%)	3 (30%)	Catering	2 (13%)	1 (7%)
Post-education	2 (14%)	1 (10%)	Management	1 (6%)	1 (7%)
			Health	5 (31%)	4 (27%)
			Non indicated	1 (6%)	1 (7%)

Respondents were either children or their parents. Results are expressed in total number and percentage

^a^Allocation order of TN = Telenursing; SC = standard care, for 12 months each

^b^Based on the harmonization of compulsory education (HarmoS) Swiss system

**Table 2:** Clinical characteristics of the study sample (children)

	Group1	Group2
	TN → SC*	SC → TN*
	(*n* = 30)	(*n* = 25)
Diagnosis		
Behçet syndrom	0	1 (4%)
JIA (enthesitis-related)	9 (30%)	7 (26%)
JIA (undifferenciated)	1 (3%)	2 (8%)
JIA (oligoarticular)	5 (17%)	10 (37%)
JIA (polyarticular)	0	4 (15%)
JIA (systemic)	1 (3%)	0
Chronic osteomyelitis	1 (3%)	1 (4%)
Auto-inflammatory disease	0 (0%)	1 (4%)
Juvenile dermatomyositis (JDM)	1 (3%)	0 (0%)
Uveitis	2 (7%)	1 (4%)
Chronic infantile neurological cutaneous articular (CINCA) syndrom	1 (3%)	0
Lupus	1 (3%)	0
Crohn’s disease	1 (3%)	0
Undetermined	4 (13%)	0
Severity ^a^		
Mild/moderate	27 (90%)	23 (92%)
Severe	3 (10%)	2 (8%)

Allocation order of *TN* telenursing; *SC* standard care, for 12 months each. *JIA* juvenile idiopathic arthritis

^a^Severity of the disease was assessed prior randomization, by the treating physician using the

JADAS

### Satisfaction

Proportions of participant who were satisfied (CSQ-8 score ≥ 30) are reported in Table 3. At T12 and T24, the interaction between the treatment and the year was not significant. A model without this interaction compared the TN and SC impact on satisfaction independently of the year it was received and showed that probability of being satisfied (satisfaction scores ≥30) was 8 times higher at the End of the TN period when compared to SC (OR = 7.7, 95% CI: 1.8–33.6). Satisfaction scores progressively increased by 20% from T0 to T12 in the TN group. An opposite negative trend was observed in the SC group, where satisfaction progressively decreased by 60% between T0 and T12.

**Table 3:** Observed proportions (%) of participants at T0, T6, T12, T18 and T24 for primary and secondary outcomes

	Intervention allocation		Proportions (%)				
Outcomes	T0 - T12	T12 -T24	T0	T6	T12	T18	T24
Satisfaction; CSQ-8 scores ≥30	TN	SC	62	60	70	58	42
	SC	TN	44	38	29	54	54
No difficulty in functional capacity ^a^	TN	SC	38	66	69	58	71
	SC	TN	42	52	54	63	64
No pain	TN	SC	70	73	85	78	82
	SC	TN	64	67	54	67	64
No morning stiffness	TN	SC	67	80	78	70	93
	SC	TN	80	71	58	79	77
Improvement of disease status ^b^	TN	SC	25	38	36	27	33
	SC	TN	23	42	18	17	9
No difficulty in physical quality of life	TN	SC	37	48	52	40	64
	SC	TN	20	46	25	46	50
No difficulty in psychosocial quality of life	TN	SC	50	59	67	60	68
	SC	TN	46	50	58	58	59

^a^Functional capacity defined as the ability to perform activities of daily living and other independent living skills

^b^Disease status is defined here by the occurrence of symptoms (absence, presence or recurrence) and course of disease from previous visit (improvement, stable or worse)

### Secondary outcomes

#### Morning stiffness

Participant’s proportions of having no morning stiffness, (item 4 of JAMAR), are reported in Table 3. At T12 and T24, there was a significant interaction between the treatment (TN or SC) and the year (*p* < 0.001) indicating a treatment carry-over from the first to the second year. Participants in group 1, who received the TN during the first year (80% without stiffness), maintained better results throughout their second year when they reversed to SC (97% with no stiffness). In contrast, participants in group 2, who started with SC (60% with no stiffness) and benefitted from the TN during their second year, maintained lower results (78% with no stiffness).

Due to this carry over treatment effect, the logistic regression analysis included the first year results only. Results indicated that the probability of having no morning stiffness would be 3 times greater after TN than after SC (OR = 3.2, 95% CI: 0.97–7.15).

#### Pain

Participant’s proportions with no pain, which rated ≤3 on the 21-point VAS (item 2 of JAMAR) are reported in Table 3.

At T12 and T24 there was a significant interaction between the treatment and the year (*p* < 0.001) indicating a treatment carry-over effect from the first to the second year. Participants in group 1, who received the TN during the first year (91% with no pain), maintained better results throughout the second year with SC (88% without pain). In contrast, participants in group 2, who started with SC (59% with no pain) and benefitted from the TN during their second year maintained lower results (67% with no pain).

Due to this carry over effect, the logistic regression analysis included the first year results only. Results pointed to an upward trend, suggesting that the probability of having no pain would be greater after TN than after SC, but this difference was not statistically significant (OR = 2.64, 95% CI: 0.97–7.15).

#### Extraarticular symptoms

Participant’s proportions with extraarticular symptoms, fever and rash, were not analysed because no more than two patients had these symptoms at any time.

#### Functional capacity

Participant’s proportions with no difficulty in their functional capacity, with a total score of 0 (item 1 of JAMAR) are reported in Table 3.

At T12 and T24, the interaction between the treatment and the year was not significant. A model without this interaction compared the impact of TN and SC on functional capacity independently of the year it was received and showed no significant differences.

#### Disease status

Participant’s proportions of improvement in disease status, who were in “remission” (item 7 of JAMAR) and for whose disease course was either “much improved” or “slightly improved” (item 8 of JAMAR) are reported in Table 3.

At T12 and T24, the interaction between the treatment and the year showed that this interaction was not significant. A model without this interaction compared the impact of TN and SC on disease status independently of the year it was received and showed no significant differences.

#### Quality of life

Participant’s proportions with no difficulty in health-related quality of life, with a total score of 0 (item 14 of JAMAR), were analyzed globally and also specifically by analyzing separately the scores of physical and psychosocial health, and are reported in Table 3.

At T12 and T24, the interaction between the treatment and the year was not significant for both physical and psychosocial quality of life. A model without this interaction compared the impact of TN and SC on physical/ psychosocial quality of life independently of the year it was received and showed no significant differences.

## Discussion

This multi-site randomized crossover study is, to the best of our knowledge, the first study demonstrating the effect of a TN intervention to support children/adolescent with inflammatory rheumatic disease and their parents. During the course of their disease, participants in the intervention group received tailored individualized affective support, health information and assistance in decision making that improved their satisfaction and impacted positively on symptoms, such as morning stiffness and pain. The intervention resulted in improvement of satisfaction, with the probability of being satisfied 8 times higher when compared to SC. Additionally, we observed that satisfaction increased by 20% at the End of the full period of the TN, whereas satisfaction decreased of 60% throughout receiving SC. This shows that as time went by, the interaction between the participant and the TN nurse increased in quality with better tailored response to individual needs and resulting in higher satisfaction with care. These results also indicate that the Cox model used in this study to conceptualize the intervention was adequate. It also highlights the importance for this type of intervention to be provided over a long period of time and regularly for the interaction to take place. Initially, this study was designed to respond to a need to fill in the gaps of a lack of follow-up between medical consultations, where parents felt they had difficulties to reach out to the appropriate person to find answers to day to day problems related to their child’s conditions. Although it concerns only a small proportion of all children attending the clinic, the intervention in this study seems to have appropriately responded to this need.

Supporting our results, satisfaction has been correlated with telehealth interventions in other studies [29–33]. Improved satisfaction is a good indicator of high-quality nursing care; a major determinant being that nurses recognize participants’ concerns and adapt their care to participants’ specific needs [34, 35]. In this study, satisfaction was the most positively impacted outcome showing that children with inflammatory rheumatic diseases and their family were appreciative of the support and information provided by the Telenursing nurse. These results are consistent with other studies performed mainly in the adult population suffering from chronic conditions such as diabetes, cancer, chronic pulmonary disease, heart failure, complex endocrinology patients or Parkinson’s disease, where Telenursing had a positive impact on several outcomes, including satisfaction [13, 29–33, 36–39]. Telehealth in the pediatric population has been less studied so far, but satisfaction and patient’s perception have been investigated. Improved communication and symptom management was demonstrated in studies with an advanced symptom management system (ASyMS©) for cancer patients [40–44], and value of convenience, confirmation, support and guidance brought by TN was showed for parents of children with gastroenteritis [18].

To a lesser extent, positive impacts on health outcomes have also been correlated with telehealth interventions [29, 30, 32, 34, 45–47]. In our study we demonstrated a positive impact of TN on morning stiffness and pain, indicating that the intervention improved symptom management. This result is in line with results obtained in other studies where health outcomes, such as metabolic control variable or symptom severity and distress have been improved notably for patients suffering from chronic conditions, such as diabetes and asthma [48, 49].

Our study had two limitations inherent to the choice of crossover design: a carryover effect of the intervention and a difference on intervention’s impact due to the sequential and temporal allocation nature of the intervention.

The carryover effect of the intervention and time impacted pain and morning stiffness outcomes. It could partly be explained by the lack of a wash-out period in our study design. However, because we had 6 months between the End of the intervention and the first measure in the SC group, providing theoretically enough time for no carry-over effect of the intervention, we concluded that a wash-out period was not necessary. Time effect of the natural course of the disease may have introduced some bias, as all participants received appropriate medical treatment that one can assume improved outcomes with time. When there was a carry-over effect, analyses were only performed in half of the data collected during the first 12 months. It must be emphasized that this type of analysis generates a decreased power in the test due to the smaller size of the sample (*n* = 24 and *n* = 28).

Another feature of the design is that all participants receive both TN and SC, and this could also explain a diluted effect of the intervention. In fact, most studies with a TN have different intervention allocation. In some studied population was divided in two and one half was only receiving TN and the other only receiving routine care [9, 10]. In another study, all patients were receiving the TN right away, and they were they own control for evaluation of outcomes, before and after intervention [37]. The aforementioned studies have shown a more significant impact in the studied outcomes most probably because they compared the full force of the intervention against no intervention or against baseline. In our study, the cross-over design was chosen because it allows for smaller sample size in a population, where the incidence of the disease is relatively small, yet its impact is significant when inappropriately managed.

The sequential allocation of the intervention impacted all outcomes. Better impact on the outcomes in the group TN receiving first as opposed to the one receiving SC first was observed. This effect has probably been exacerbated by the participant inclusion criterion of newly diagnosed patients only. It is known that newly-diagnosed patients with complex healthcare needs require close monitoring and time to adjust to the diagnosis, constraints of the treatment, and to cope with doubts and uncertainty for the future [33]. Participants receiving the TN first were likely to require more support and help than those who received the intervention minimum one year after diagnosis. This could explain better results in group 1. However, the positive impact in group 2 should not be overlooked; albeit diminished, it was still indicating usefulness of Telenursing in the long term.

Finally, because blinding of participants was not possible in this study, it introduced potential biais in participants’ self-reported outcomes. Power calculation was performed on the primary outcome only, therefore results related to secondary outcomes should be interpreted with caution. Further testing and economic evaluation are warranted prior to implementation into practice.

## Conclusions

In summary, our Telenursing intervention combined affective support, health information and assistance in decision making in a new and effective approach. Patient with inflammatory rheumatic diseases and family were satisfied, and children tended to have less morning stiffness and pain. This nurse-led telephone intervention has the potential to reduce health problems, whilst increasing patients’ and family’s satisfaction during the management of chronic, debilitating pediatrics rheumatic disease, especially when administered in the newly-diagnosis period.

## Abbreviations

CINCA: Chronic infantile neurological cutaneous articular
CSQ-8: Client satisfaction questionnaire-8
ERA: Enthesitis-related
IMCHB: Interaction model of client health behavior
JADAS: Juvenile arthritis diseases activity score
JAMAR: Juvenile arthritis multidimensional assessment report
JDM: Juvenile dermatomyositis
JIA: Juvenile idiopathic arthritis
SC: Standard care
TN: Telenursing
VAS: Visual analogue scale
